# BlastoDB: first release of a community-driven multi-omics and epidemiological resource for *Blastocystis* biology and subtyping

**DOI:** 10.12688/openreseurope.23235.2

**Published:** 2026-05-18

**Authors:** Iliya Dauda Kwoji, William Edwards, Abbey Ruffell, Daisy Shaw, Constance Denoyelle, Ana Figuiredo, Isabel Guadano-Procesi, Jaya Makkimane, Kalliopi Pantzi, Anja Godfrey, Eleni Gentekaki, Christen Rune Stensvold, Martin Kolisko, Anastasios Tsaousis

**Affiliations:** 1 Department of Medical Microbiology, Second Faculty of Medicine, Charles University, Prague, Czech Republic; 2 University of Kent School of Biosciences, Canterbury, England, UK; 3 CNRS, Inserm, CHU Lille, Institut Pasteur de Lille, University of Lille, Lille, France; 4 Department of Biology and CESAM, University of Aveiro, Aveiro, Portugal; 5 Department of Clinical Science and Translational Medicine, Tor Vergata University of Rome, Rome, Italy; 6 Department of Veterinary Medicine, University of Nicosia, Nicosia, Nicosia, Cyprus; 7 Laboratory of Parasitology, Department of Bacteria, Parasites, and Fungi, Statens Serum Institut, Copenhagen, Denmark; 8 Czech Academy of Sciences Czech Centre for Phenogenomics, Vestec, Central Bohemian Region, Czech Republic

**Keywords:** Blastocystis; database; epidemiology; microbiome; multi-omics; subtypes; community resource; One Health

## Abstract

BlastoDB ( https://www.blastodb.com/) is developed as an open-access, community-driven resource dedicated to *Blastocystis*, one of the most common yet understudied intestinal protists. BlastoDB will offer the scientific community up-to-date, curated information on *Blastocystis* by integrating epidemiological data, microbiome profiles, multi-omics datasets (genomics, transcriptomics, proteomics, and metabolomics), reference sequences for subtypes, protocols, microscopy images, and related metadata. In this initial release, we describe the data model, database architecture, curation pipelines, and web interface, which together facilitate subtype classification, comparative and integrative analyses, and cross-study synthesis of epidemiological and experimental data. We outline submission and governance workflows designed to support community contributions, training activities, and sustainable curation under the “ *Blastocystis* under One Health” COST Action (CA21105). Finally, we highlight planned extensions, including expanded metagenomic and metatranscriptomic content, automated genome quality assessments, metagenome-assembled genomes, and geospatial and analytical dashboards. BlastoDB provides a central, FAIR-aligned hub for *Blastocystis* data, images, and protocols, reducing technical barriers and fostering a collaborative ecosystem for studying this globally prevalent protist.

## 1. Introduction

*Blastocystis* is a single-celled microbial eukaryote and the most common intestinal protist in human faeces. ^1^ Although *Blastocystis* is frequently detected in epidemiological studies, its biology, epidemiology, and clinical significance remain incompletely understood. ^2^ The field is shaped by recurring issues: a lack of unified nomenclature, inconsistent diagnostic methods, uneven and inconsistent reporting of datasets and associated metadata. These factors significantly impede cross-study comparison and synthesis. ^3^

Several platforms currently host *Blastocystis*-related data, including the PubMLST *Blastocystis* platform ( https://pubmlst.org/organisms/blastocystis-spp ), an 18S rRNA non-redundant reference set, NCBI, where a small number of reference genomes are deposited, and other sequence repositories. ^3^ However, these resources are typically centred on single marker genes or highly fragmented genome scale datasets. There are few well assembled and annotated genomes. ^4– 7^ Regardless, almost none of the resources provide integrated multi-omics and epidemiological content with harmonised metadata ( Table 1). As multi-omics datasets (genomic, transcriptomic, proteomic, metabolomic) integrating microbiome data ^8, 9^ and clinical/epidemiological information accumulate, ^10, 11^ there is a clear need for a curated, subtype-aware, and community-oriented resource that consolidates these datasets.

**Table 1.** Current databases and links with *Blastocystis* data.

Database/link	Scope	Datatype	Strengths	Drawbacks	Reference
PubMLST – *Blastocystis* subtype/MLST database	Typing and allele/ST annotations	18S rRNA and MLST alleles, STs, isolate metadata	Gold standard for subtype classification	Focused mainly on marker gene	^3^
Zenodo 18S non-redundant database	18S rRNA reference dataset	Curated 18S rRNA Fasta	Ready-to-use reference for amplicon pipelines	Single locus only	^13^
Genoscope Genome Browser	Reference genome browser	Genome assemblies and annotations	Gene-level browser for reference genomes	Few genomes	^14^
NCBI (GenBank/Genome/SRA)	General sequence repository	Genomes, raw reads, transcriptomes	Largest sequence database	Fragmented annotations, mostly non-curated	^7^

BlastoDB will be created as a Bioinformatic Resource Hub (BRH) by the “ *Blastocystis* under One Health” COST Action, funded by the European Union's European Cooperation in Science and Technology (COST) programme. ^12^ The database is designed to enhance global understanding of *Blastocystis* within a One Health approach and to provide a single environment that can host and integrate different kinds of data on this often-overlooked gut protist and allow sharing with the scientific community. BlastoDB achieves this through the curation of subtype sequences, multi-omics datasets, culture and microscopic features, and by tackling several scientific challenges in *Blastocystis* research, including data source heterogeneity, lack of standardisation, underutilisation of available metadata, and fragmentated community.

The objectives of BlastoDB are to consolidate subtype reference sequences and genomes, collate epidemiological and microbiome datasets, offer a simple interface for querying and visualising data, improve metadata standards, and encourage community contributions. ^12^ BlastoDB aims to harmonise different omics layers, including genomics, transcriptomics, proteomics, and metabolomics, and to connect these with epidemiological and microbiome information, protocols, and visual resources. The long-term goal is to support a comprehensive understanding of the role of *Blastocystis* in health and disease across hosts and environments.

In this manuscript, we introduce the first public release of BlastoDB. We detail its content, structure and curation, the main interface, and key use cases. We then consider how BlastoDB can develop into a comprehensive, sustainable reference for *Blastocystis* research.

## 2. BlastoDB, an integrated multi-omics and epidemiological resource

**Figure 1.**
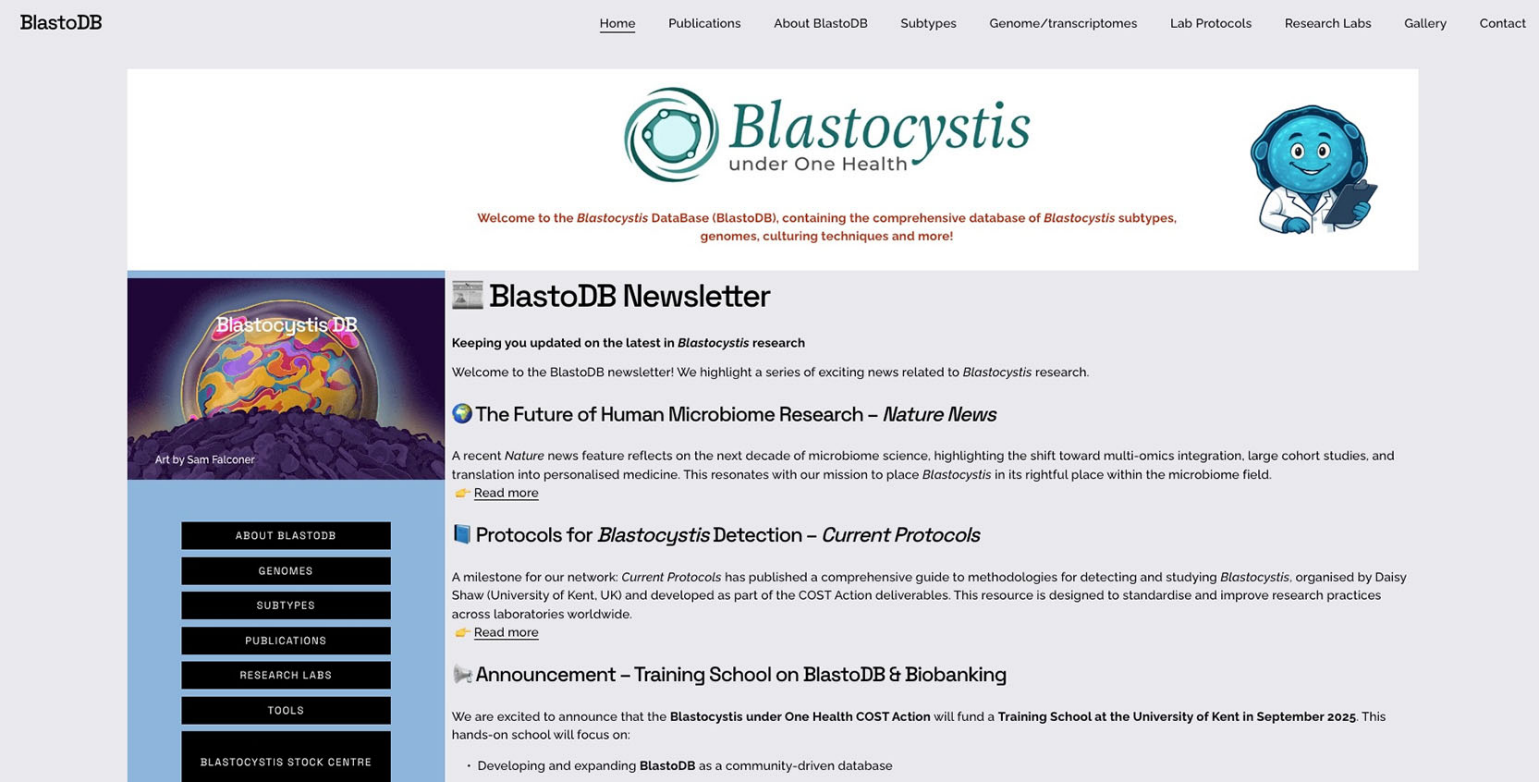
BlastoDB homepage. Screenshot of the BlastoDB homepage ( https://www.blastodb.com/). The main navigation bar provides access to Subtypes, Genomes and transcriptomes, Data and protocols, and Labs and resources. A global search bar supports keyword queries across subtypes, hosts, locations and studies. Highlighted panels give quick access to featured datasets, recent updates and documentation.

BlastoDB will be a user-friendly, open-access resource with simple, click-and-go navigation. The main utilities are accessible from the homepage by selecting the relevant sections ( Figure 1). This first release focuses on making curated data and key tools available, while establishing the framework for future expansion.

### 2.1 Database content and structure

**Figure 2.**
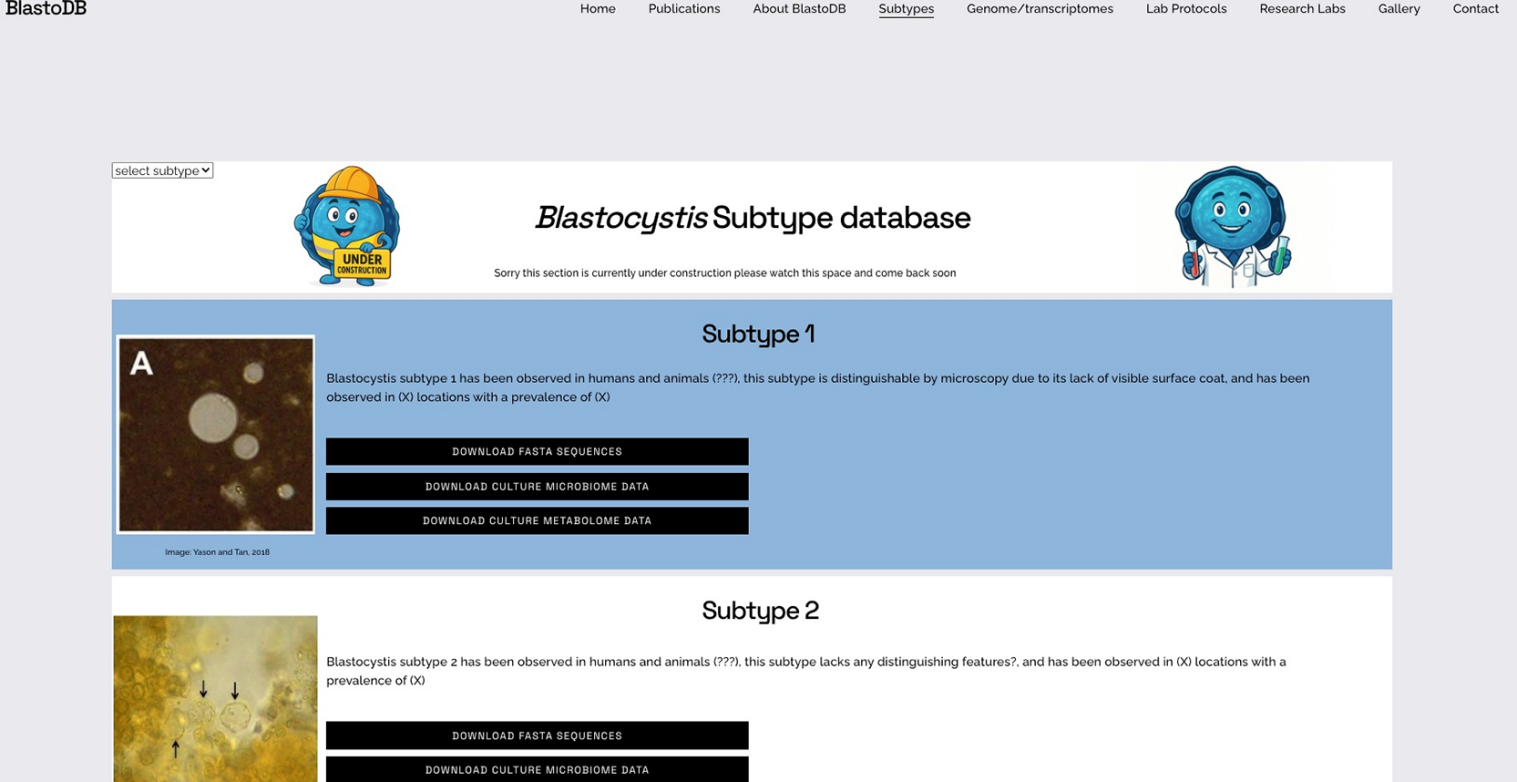
Example subtype page in BlastoDB. Representative subtype page illustrating the integration of marker sequences, genomes and metadata. The page summarises host range and geographic distribution for the subtype, lists curated marker sequences with download links, and displays available genomes and transcriptomes. Links to culture-derived multi-omics datasets, protocols and relevant laboratories are shown in dedicated sections.

BlastoDB covers a broad set of data types and resources:

• **Subtype reference sequences and markers**
  Curated *SSU* rRNA and other marker sequences with subtype assignments, including links to external schemes and reference sets where applicable. These form the backbone for subtype classification and phylogenetic analyses. For each subtype assignment, BlastoDB records the amplified region of the 18S rRNA gene, the primers used, and the sequencing strategy employed (e.g., Sanger sequencing or amplicon-based next-generation sequencing), enabling users to assess classification consistency and comparability across studies.
• **Genomes and transcriptomes**
  Raw reads and assembled genomes and transcriptomes from cultured isolates, with annotations where available. These datasets will be linked to isolates, subtypes, hosts and studies.
• **Microbiome and other multi-omics datasets**
  Culture-derived and host-associated datasets, including bacterial microbiome profiles, metabolomics and other omics outputs. Each dataset will be linked to subtype, host, sample type, culture conditions and any experimental (published) protocols. For 16S rRNA-based microbiome datasets, the sequencing platform (e.g., Illumina-based technologies), the amplified 16S rRNA gene region (e.g., V3–V4), and the DNA extraction method are captured as mandatory metadata fields, given their substantial influence on observed microbial community composition and cross-study comparability.
• **Epidemiological and clinical metadata**
  Host taxonomy, geographic information (country, region), sampling context (community, hospital, cohort study), clinical phenotypes (e.g. symptomatic vs asymptomatic in the gut) and core methodological descriptors (sample type, extraction protocol, sequencing platform, amplified gene region, and primer set).
• **Protocols and laboratory resources**
  Standard operating procedures for microscopy, molecular detection, culturing and subtyping, as well as links to *Blastocystis* culture collections and laboratories. This includes information on the laboratories on where the characterised isolates and specific subtypes can be obtained.
• **Images and visual resources**
  Microscopy images from wet mounts and culture illustrating *Blastocystis* morphology will be linked with life cycle stages, subtypes, isolates, protocols (e.g. culture conditions) and datasets ( Figure 2).

Metadata standards in BlastoDB include host taxonomy, geographic information, clinical phenotypes and methodological details. These will be stored in a database structure that connects isolates, sequences, experiments, protocols, laboratories, images and publications. The architecture combines this relational backend with a set of REST-style APIs and a browser-based frontend.

### 2.2 Data acquisition and curation

BlastoDB will integrate data from three primary sources:

1. **Public repositories**
  Genome assemblies, transcriptomes, marker sequences, and selected microbiome or metabolomic datasets are collected from GenBank/ENA/SRA, GEO, and related repositories using *Blastocystis*-specific queries and manual screening.
2. **Published supplements**
  Detailed metadata, epidemiological information, and multi-omics measurements that are not readily accessible in public data archives are extracted from the supplementary materials of key *Blastocystis* studies. The minimum requirements for these depositories will be evaluated upon collection of all the data.
3. **Community contributions**
  Researchers can directly submit new (raw or assembled) data to BlastoDB, including genomes, marker sequences, epidemiological datasets, microbiome profiles, culture observations, protocols, and images. Submission templates, validation rules, and documentation are available on the website. Where possible, submitters are encouraged to deposit primary data in long-term repositories (e.g., sequence archives, Zenodo) and link these to BlastoDB using accession numbers or DOIs.

Incoming data pass through a curation pipeline with the following stages:

• Ingestion and basic format validation;
• Mapping to the internal data model;
• Metadata normalisation and alignment to controlled vocabularies (e.g. host taxonomy, country codes);
• Subtype assignment and verification using curated marker reference sets and classifiers;
• Quality checks for genomes, transcriptomes and multi-omics data when relevant metrics are available; and
• Manual review of flagged records and ambiguous subtype calls.

Records will be given curation status labels (e.g. provisional, curated, deprecated). Curated entries are prioritised in searches, exports and subtype summaries.

Community contributions are encouraged through clear submission guidelines, DOI-aware workflows, and feedback mechanisms. Contributors are recognised in the database and in release notes.

### 2.3 Implementation and architecture

BlastoDB’s backend is currently built as a relational database that stores key entities (isolates, sequences, omics datasets, epidemiological records, protocols, laboratories, images) and their interconnections. The database is accessed via a set of APIs that offer:

• Search and filter operations for isolates, subtypes, hosts, datasets and protocols;
• Programmatic retrieval of data in machine-readable formats (e.g. JSON, TSV, FASTA); and
• Endpoints for sequence classification and related utilities.

The web interface is built using a modern JavaScript framework. It consumes APIs and displays users with interactive tables, subtype pages, dataset summaries, and visualisation components. The system is deployed as containerised services behind a reverse proxy, which simplifies updates and scaling.

Each major content update will be treated as a release, with a release date, summary of changes and archived exports. This supports reproducible analyses and allows users to track changes in content and schema over time.

### 2.4 Functionalities, tools and visual resources

BlastoDB offers several tools and views to help users explore and use the data:

• **Homepage navigation**
  The homepage provides direct entry points to Subtypes, Genomes and transcriptomes, Data and protocols, and Labs and resources, along with news on recent releases and featured datasets ( Figure 1).
• **Search and filtering**
  A global search bar supports keyword searches across subtype names, hosts, countries, studies and data types. Results can be filtered using facets such as subtype, host species, country, data type or year and exported as tables or FASTA files.
• **Subtype pages**
  For each recognised subtype, BlastoDB provides a summary page with:
  ○ Host range and geographic distribution where known;
  ○ Curated marker sequences and download links;
  ○ Associated genomes, transcriptomes and other omics datasets;
  ○ Key epidemiological and clinical information;
  ○ Linked protocols and reference laboratories; and
  ○ Representative microscopy images and culture photographs.
• **Genome and transcriptome pages**
  These pages will contain assembly metrics where available, isolate metadata (subtype, host, country, sampling context), links to external genome browsers or repositories, and cross-links to relevant subtype and dataset pages.
• **Sequence classifier and alignment tools**
  Users can submit marker sequences (for example, *SSU* rRNA amplicons) to obtain subtype assignments with similarity scores and links to the closest reference sequences. Basic alignment and tree visualisation tools allow quick exploration of relationships among reference and query sequences.
• **Downloads and programmatic access**
  Batch downloads of marker sets, genome collections or curated metadata can be triggered through the website. Programmatic users can access the same data via documented API endpoints.
• **Community and training sections**
  Dedicated pages that list laboratories, culture collections, available protocols and training materials, as well as information on COST training schools and short-term scientific missions relevant to *Blastocystis.*

These tools together support tasks ranging from routine subtype assignment and dataset discovery to more elaborate meta-analyses and hypothesis generation.

## 3. Example use cases

This section illustrates how BlastoDB can be used in practice. The specific datasets and numbers can be adapted to real examples when you finalise the manuscript.

### 3.1 Subtype assignment and contextualisation of a new cohort

A research group performs *SSU* rRNA amplicon sequencing in a human cohort and wants to determine the distribution of *Blastocystis* subtypes and place their findings in context.

1. Representative *SSU* rRNA sequences are submitted to the BlastoDB sequence classifier.
2. The classifier returns subtype assignments with similarity scores and links to closest reference sequences.
3. Navigation to the subtype pages to review known host ranges and geographic distributions and to download curated reference FASTA files for pipeline validation.
4. Using the genomes and transcriptomes section, identifation of available genome resources for the dominant subtypes in the research group’s cohort.
5. Export OF subtype- and host-specific metadata for comparative analyses and for planning follow-up work, such as culture attempts or targeted metabolomics.

BlastoDB therefore will provide a single environment for subtype assignment, reference selection and basic epidemiological contextualisation.

### 3.2 Comparing subtype distributions across hosts and regions

Another researcher is interested in how *Blastocystis* subtypes are distributed across human and animal hosts in different regions and take the following steps:

1. Search BlastoDB for all curated entries with subtype assignments and associated host and country metadata.
2. Filters are used to select the relevant host groups (for example humans, cattle, companion animals) and regions.
3. The filtered result table, including subtype, host, country, study and key methodological fields, is exported.
4. In an external statistical environment, subtype–host–region associations are analysed and differences in subtype prevalence between settings are tested for.

BlastoDB will streamline the process of discovering, harmonising and exporting data suitable for these analyses.

## 4. Discussion and future directions

In this initial release, BlastoDB tackles several longstanding bottlenecks in *Blastocystis* research. It consolidates curated subtype reference sequences, genomes, transcriptomes, proteomes, microbiome profiles, metabolomics, and other multi-omics datasets, along with epidemiological metadata, protocols, laboratory information, and visual resources. This integrated structure will enable researchers to explore more easily the connections between molecular diversity, epidemiology, microbiome context, and experimental biology.

By integrating standardised protocols, culture resources, and subtyping schemes within the same environment as the data, BlastoDB begins to address the methodological heterogeneity that has restricted cross-study comparability. It facilitates routine tasks such as subtype assignment and dataset discovery, while also lowering barriers to more advanced research, including strain-resolved comparative genomics, subtype-specific association studies, and combined analyses of host, microbiome, and parasite features. The inclusion of microscopy images and culture photographs adds an essential visual dimension, helping to link in silico and in vitro perspectives.

**Figure 3.**
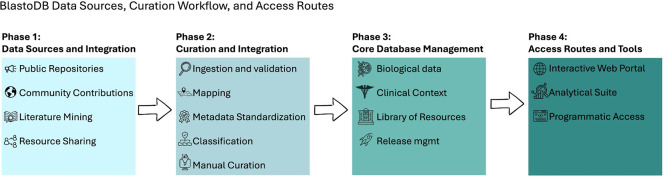
Schematic overview of the BlastoDB data flow and architecture. Data are collected from three main sources: public repositories (GenBank/ENA/SRA/GEO), published studies and supplementary materials, and direct community submissions. Incoming records pass through an ingestion and curation pipeline comprising basic validation, mapping to the internal data model, metadata standardisation, subtype assignment and quality control, and manual review. Curated entries are stored in the BlastoDB core database, which integrates isolates and subtypes, epidemiological and clinical metadata, microbiome and other multi-omics datasets, protocols, laboratory information and images. Versioned releases and documentation are generated from this core. Users access BlastoDB through a web interface (subtype and genome/transcriptome pages, data and protocol browser, image resources), built-in analysis tools (sequence classifier, alignment and tree viewers, filtered downloads) and programmatic access via APIs and release snapshots.

BlastoDB is, by design, a community-driven ecosystem rather than a static catalogue. Submissions of all types from researchers worldwide are welcomed, including new genomes, subtype sequences, epidemiological datasets, microbiome data, culture observations, protocols, images, and additional metadata annotations. Clear submission guidelines and DOI-aware workflows are intended to keep the resource aligned with FAIR principles while allowing it to expand in scope and geographic coverage ( Figure 3). By integrating batch search tools, sequence classification, alignment visualisation, and basic phylogenetic exploration, BlastoDB reduces technical barriers for both novice and experienced users and enhances global capacity for *Blastocystis* research.

Sustainability remains a challenge. Long-term curation and infrastructure require stable funding and dedicated curator time. Embedding BlastoDB within the “ *Blastocystis* under One Health” COST Action ^12^ and its working groups offers a strategic advantage by providing access to training activities, governance structures, and a large, engaged community. Future collaborations with microbiome consortia, pathogen databases, and generic data infrastructures can further enhance sustainability and interoperability. Over time, we aim to nurture an active “usage ecology” around BlastoDB by providing accessible training and outreach materials, including video-based tutorials and worked examples that illustrate typical workflows and best practices.

Looking ahead, there are several clear directions for development:

○ Incorporation of metagenomic and metatranscriptomic datasets, including metagenome-assembled genomes and more detailed host and microbiome context;
○ Automated genome quality assessment and improved tools for detecting contamination and misassembles;
○ Geospatial dashboards for interactive visualisation of subtype distributions, hosts, and environmental contexts;
○ Analytical modules for integrating multi-omics, microbiome, and clinical data, including machine-learning approaches to explore host–parasite interactions; and
○ Richer, interactive comparative genomics environments that enable users to build and share in-browser analyses.
○ AI-assisted curation and discovery tools that can harmonise and enrich metadata, flag inconsistencies, propose subtype assignments, and recommend relevant datasets, protocols and virtual biobank samples based on user queries and patterns in the data

Taken together, these additions will transform BlastoDB into a more dynamic platform. As data production speeds up across human, animal, and environmental interfaces, BlastoDB will offer a framework to integrate diverse datasets, facilitate reproducible research, and generate new insights into *Blastocystis* biology, its interactions with hosts, and its potential roles in health and disease.

## Software availability

BlastoDB is provided as a hosted web application and is accessible via a standard web browser. At the time of publication there is no separate standalone software package or installable version of BlastoDB, and the underlying Squarespace-based implementation is not available as an independent open-source codebase. All functionality described in this article is accessible through the online platform. No additional software is required to use BlastoDB beyond a web browser and internet connection.

## Ethics

Ethical approval and consent were not required for this article because it does not report any new studies involving human participants or animals. The work describes the design and implementation of a database resource that collates previously published and publicly available datasets.

## Data Availability

No new data are associated with this article. BlastoDB is an online resource that integrates previously published and publicly available datasets, which are cited in the main text and reference list. All underlying data remain available from their original repositories under the access conditions described in the cited publications.
