# Supplementary material for: BlastoDB: first release of a community-driven multi-omics and epidemiological resource for Blastocystis biology and subtyping
Source: Open Res Eur. 2026 May 18;6:65. Originally published 2026 Mar 4. [Version 2] doi: 10.12688/openreseurope.23235.2 (PMC13033127; doi:10.12688/openreseurope.23235.2)
Supplement: Peer review history

# Reviewer report: Reviewer response for version 2

**DOI:** 10.21956/openreseurope.25738.r74610

**Published:** 2026-05-27

**Authors:** Małgorzata Lepczyńska

**Affiliations:** 1 Department of Medical Biology, School of Public Health, Collegium Medicum, University of Warmia and Mazury in Olsztyn, Olsztyn, Poland

The manuscript is approved.

Is the rationale for developing the new software tool clearly explained?

Yes

Is the description of the software tool technically sound?

Yes

Are sufficient details of the code, methods and analysis (if applicable) provided to allow replication of the software development and its use by others?

Yes

Is sufficient information provided to allow interpretation of the expected output datasets and any results generated using the tool?

Yes

Reviewer Expertise:

Medical Microbiology and Parasitology (especially Blastocystis)

I confirm that I have read this submission and believe that I have an appropriate level of expertise to confirm that it is of an acceptable scientific standard.

# Reviewer report: Reviewer response for version 1

**DOI:** 10.21956/openreseurope.25139.r70747

**Published:** 2026-03-28

**Authors:** Małgorzata Lepczyńska

**Affiliations:** 1 Department of Medical Biology, School of Public Health, Collegium Medicum, University of Warmia and Mazury in Olsztyn, Olsztyn, Poland

This manuscript presents a new valuable database for conducting reliable research on *Blastocystis*, beginning with properly collecting the samples and ending with the interpretation of molecular examination results. The focus on *setting up a *database *storing isolates, sequences, experiments, protocols, laboratories, images, and publications together *is extremely relevant, considering the current doubtful published epidemiological and molecular data of *Blastocystis* subtypes, their prevalence, and discoveries of the new ones. The authors present a new platform that aims both to collect reliable information on *Blastocystis* and to verify existing data, which will help avoid mistakes in identifying *Blastocystis* subtypes by researchers all over the world. It will be possible especially by using verified subtype reference sequences and genomes connected with epidemiological and microbiome information, protocols, and other resources.

 The introduction of the manuscript highlights the difficulties in finding appropriate tools for analyzing genetic sequences from which *Blastocystis* subtypes can be confidently determined, which have become a challenge in recent years. The growing disinformation regarding the interpretation of molecular test results is increasingly irritating. Compared to the existing, similar tools, BlastoDB is the first curated, reliable, and comprehensive resource that combines multi-omics datasets (genomic, transcriptomic, proteomic, and metabolomic) accumulated for years by publishing the microbiome data as well as clinical/epidemiological information in one place. The authors mention a few websites/platforms where *Blastocystis* is one of many organisms described and the information about it is incomplete or fragmentary. According to that, almost none of the resources provide integrated content with high-quality metadata for comparison and successful reuse of data. BlastoDB provides a significant advance over previously published software.

 The software is an open-access resource, freely available for users for non-commercial purposes, with simple navigation. It works as the authors described. BlastoDB offers several tools and views to help users explore and use the data. It adequately links the information necessary to investigate the genetic diversity of the protist and its epidemiology by the process of gathering, cleaning, and merging data from multiple, disparate sources into a single, centralized repository. It improves data quality, reduces redundancy, and enables efficient analysis by creating a "single source of truth." The authors of this article invite researchers to directly submit new data to the platform, making available on the website submission templates, validation rules, and documentation. The database content and structure are well described and presented on the figures as screenshots of the BlastoDB homepage and example subtype page, which is a helpful addition that makes it more accessible and clear. The authors describe in detail the purpose of the tabs on the website, how to use them, how to interpret the results obtained there, and how to use this data for further analysis. The section named “Example use cases” shows to the reader how the platform can be used in practice, which strengthens their understanding and demonstrates the software's functionality. The data’s primary sources were chosen very carefully and passed through a multi-stage curation pipeline. In addition, the selection process is clearly described and explained. The chosen documentation is sufficient to run the tool. The big advantage is that the authors state that there is no separate software package or installable version of BlastoDB, and all functions described are accessible online through a web browser and internet connection.

 This manuscript introduces to the reader BlastoDB, a new software that can help develop methodologies for the detection and subtyping of *Blastocystis* so the research findings can be more reliable than the results published previously and additionally establishes a *Blastocystis* subtype and microbiome databank to make new research easier for all scientists as well as consolidate existing data. In general, the article is well written and structured. Each part of the manuscript presents the content clearly and orderly. The manuscript presents the assumptions of the created platform in an accessible and concise way, emphasizing the importance of collecting different types of data in one place.

Is the rationale for developing the new software tool clearly explained?

Yes

Is the description of the software tool technically sound?

Yes

Are sufficient details of the code, methods and analysis (if applicable) provided to allow replication of the software development and its use by others?

Yes

Is sufficient information provided to allow interpretation of the expected output datasets and any results generated using the tool?

Yes

Reviewer Expertise:

Medical Microbiology and Parasitology (especially Blastocystis)

I confirm that I have read this submission and believe that I have an appropriate level of expertise to confirm that it is of an acceptable scientific standard.

# Response

**Published:** 2026-04-21

**Authors:** Anastasios Tsaousis

**Affiliations:** School of Biosciences, University of Kent, Canterbury, England, UK

We are grateful to the reviewer for her comprehensive and highly positive assessment of the manuscript. Her detailed reading of BlastoDB’s rationale, design, curation pipeline, and use cases is greatly appreciated. We are pleased that she finds the platform to be a significant advance over previously available resources and that she considers the manuscript well written and clearly structured. She raises no specific concerns requiring changes to the manuscript. We note, however, that several of the strengths she highlights—particularly the importance of reliable metadata for subtype classification and the need for a curated, community-driven hub—are precisely the areas further strengthened by the revisions made in response to Reviewer 1’s comments (see above). We believe these additions complement and reinforce the aims described in the manuscript. No changes to the manuscript were made in direct response to Reviewer 2’s comments beyond those already described above.

# Reviewer report: Reviewer response for version 1

**DOI:** 10.21956/openreseurope.25139.r70752

**Published:** 2026-03-27

**Authors:** Liliana Morales

**Affiliations:** 1 Universidad El Bosque, Bogotá, Colombia

The article provides a detailed description of the structure and organization of the BlastoDB platform, as well as its various applications for integrating and standardizing information on one of the most prevalent intestinal protozoa worldwide. However, certain aspects can be strengthened to improve the clarity and reproducibility of the data presentation.

 It is important to note that the assignment of subtypes (STs) in Blastocystis depends largely on the region of the 18S rRNA gene analyzed and the sequencing strategy employed.

 In recent years, different regions of this gene (e.g., the “barcode” region vs. partial regions) and different methodological approaches (Sanger sequencing, amplicon-based NGS) have been used, leading to inconsistencies in classification and comparability across studies. For this reason, I suggest that information regarding the amplified region, the primers used, and the sequencing strategy be explicitly included alongside the subtype assignment.

 Furthermore, the analysis of microbiomes associated with Blastocystis is a highly relevant component. In this context, it would be advisable for the sequencing platform used for microbiome analysis (e.g., Illumina-based technologies), as well as the amplified 16S rRNA gene region and DNA extraction methods, to be included alongside the reported data, given that these factors can introduce significant biases in the observed microbial composition and affect comparability between studies.

Is the rationale for developing the new software tool clearly explained?

Yes

Is the description of the software tool technically sound?

Yes

Are sufficient details of the code, methods and analysis (if applicable) provided to allow replication of the software development and its use by others?

Partly

Is sufficient information provided to allow interpretation of the expected output datasets and any results generated using the tool?

Yes

Reviewer Expertise:

Molecular parasitology. Molecular and microscopic diagnosis of intestinal parasites.

I confirm that I have read this submission and believe that I have an appropriate level of expertise to confirm that it is of an acceptable scientific standard.

# Response

**Published:** 2026-04-21

**Authors:** Anastasios Tsaousis

**Affiliations:** School of Biosciences, University of Kent, Canterbury, England, UK

We thank the reviewer for her thorough and constructive feedback. Her comments identify important aspects of methodological transparency that are central to the mission of BlastoDB. We have addressed each point in full below, and the corresponding revisions are reflected in the tracked-changes version of the manuscript.

**Comment 1.1: Subtype assignment and 18S rRNA gene region/sequencing strategy**

**Reviewer comment: **"It is important to note that the assignment of subtypes (STs) in *Blastocystis* depends largely on the region of the 18S rRNA gene analyzed and the sequencing strategy employed. In recent years, different regions of this gene (e.g., the 'barcode' region vs. partial regions) and different methodological approaches (Sanger sequencing, amplicon-based NGS) have been used, leading to inconsistencies in classification and comparability across studies. For this reason, I suggest that information regarding the amplified region, the primers used, and the sequencing strategy be explicitly included alongside the subtype assignment."

**Response: **We fully agree with this important observation. Inconsistencies in the region of the 18S rRNA gene amplified and in the sequencing strategy used are well-recognised sources of subtype misclassification and cross-study incomparability. We have therefore revised the description of the **Subtype reference sequences and markers** content category in Section 2.1 to make explicit that BlastoDB records the amplified region of the 18S rRNA gene, the primers used, and the sequencing strategy (e.g., Sanger sequencing or amplicon-based NGS) alongside each subtype assignment. Specifically, the following sentence has been added: “For each subtype assignment, BlastoDB records the amplified region of the 18S rRNA gene, the primers used, and the sequencing strategy employed (e.g., Sanger sequencing or amplicon-based next-generation sequencing), enabling users to assess classification consistency and comparability across studies.” These fields are treated as mandatory metadata in the BlastoDB data model, consistent with the database’s broader goal of enabling rigorous cross-study synthesis.

**Comment 1.2: Microbiome dataset metadata (sequencing platform, 16S rRNA region, DNA extraction)** **Reviewer comment: **"The analysis of microbiomes associated with *Blastocystis* is a highly relevant component. In this context, it would be advisable for the sequencing platform used for microbiome analysis (e.g., Illumina-based technologies), as well as the amplified 16S rRNA gene region and DNA extraction methods, to be included alongside the reported data, given that these factors can introduce significant biases in the observed microbial composition and affect comparability between studies."

**Response: **We thank the reviewer for raising this point. It is well established that choice of sequencing platform, amplified 16S rRNA gene region (e.g., V3–V4), and DNA extraction method all substantially influence observed microbial community composition, and their omission from metadata makes cross-study comparisons unreliable. We have revised the description of the **Microbiome and other multi-omics datasets** content category in Section 2.1 to specify that these three parameters are captured as mandatory metadata fields for 16S rRNA-based microbiome datasets. The following sentence has been added: “For 16S rRNA-based microbiome datasets, the sequencing platform (e.g., Illumina-based technologies), the amplified 16S rRNA gene region (e.g., V3–V4), and the DNA extraction method are captured as mandatory metadata fields, given their substantial influence on observed microbial community composition and cross-study comparability.” Furthermore, we have updated the description of the **Epidemiological and clinical metadata** category in the same section to expand the list of core methodological descriptors to include “amplified gene region” and “primer set” alongside sample type, extraction protocol, and sequencing platform. This ensures that these fields are consistently surfaced across all relevant metadata contexts within the database.
